# Association between Life’s Essential 8 and Atherogenic Index of Plasma in adults: insights from NHANES 2007–2018

**DOI:** 10.3389/fendo.2025.1506884

**Published:** 2025-02-18

**Authors:** Long-Hui Xu, Kai-Wen Ding, Guo-Dong Yang, Xiao-Xuan Han, Xiao Cong, Rong-Hui Wang, Xin-Ru Liu, Na Li, Cui-Ping Xu

**Affiliations:** ^1^ School of Nursing, Shandong University of Traditional Chinese Medicine, Jinan, Shandong, China; ^2^ Department of Nursing, The First Affiliated Hospital of Shandong First Medical University and Shandong Provincial Qianfoshan Hospital, Jinan, Shandong, China; ^3^ Department of Clinical Pharmacy, The First Affiliated Hospital of Shandong First Medical University and Shandong Provincial Qianfoshan Hospital, Jinan, Shandong, China

**Keywords:** Life’s Essential 8, Atherogenic Index of Plasma, cardiovascular health, cardiovascular disease, NHANES

## Abstract

**Introduction:**

Cardiovascular Disease (CVD) has become a significant global public health challenge, contributing to rising mortality rates. This study aims to investigate the relationship between Life’s Essential 8 (LE8) and the Atherogenic Index of Plasma (AIP), providing insights into the assessment and improvement of Cardiovascular Health (CVH).

**Methods:**

We conducted an analysis of data from 8,215 U.S. adults aged 20 years and older, utilizing the National Health and Nutrition Examination Survey (NHANES) data from 2007 to 2018. Based on the LE8 score, CVH was classified into three levels—low, moderate, and high—while AIP was classified into four risk levels: extremely low (AIP<-0.3), low (-0.3≤AIP<0.1), medium (0.1≤AIP<0.24), and high (AIP≥0.24). Weighted ordinal logistic regression analysis was utilized to examine the association between the LE8 score and the AIP risk level, adjusting for potential confounding variables.

**Results:**

A significant negative correlation exists between the LE8 score and the AIP risk level (*OR*=0.51, *95%CI*: 0.49-0.54, *P*<0.001). Higher CVH were associated with lower AIP risk levels, while lower CVH corresponded to elevated AIP risk levels. Notably, improvements in specific LE8 components—such as body mass index and blood lipids—exhibited a strong relationship with reductions in the AIP risk level.

**Discussion:**

This study suggests that the LE8 may serve as a preventive factor for CVD risk and implies that individuals can actively regulate their metabolic characteristics by optimizing their lifestyle.

## Graphical Abstract

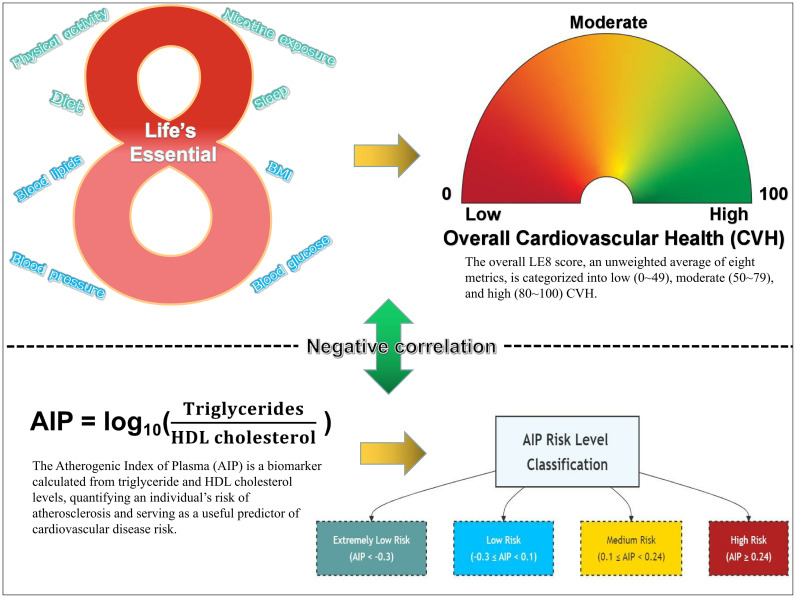

## 1 Introduction

Cardiovascular Disease (CVD), primarily encompassing ischemic heart disease and stroke, has emerged as an increasingly severe public health challenge on a global scale (1). According to statistical data, the number of deaths attributable to CVD worldwide rose from 12.4 million in 1990 to 19.8 million in 2022, with Eastern Europe exhibiting the highest age-standardized mortality rate, reaching 553 deaths per 100,000 individuals (2, 3). In light of this alarming trend, the American Heart Association (AHA) formally introduced the concept of Cardiovascular Health (CVH) in 2010, aimed at evaluating and enhancing cardiovascular well-being among individuals and populations by quantifying specific health metrics. This initiative seeks to mitigate the risk of CVD, improve quality of life, extend longevity, reduce healthcare expenditures, and foster public health (4, 5). The initial framework of CVH, referred to as “Life’s Simple 7” (LS7), comprises seven principal cardiovascular risk factors: diet, physical activity, current smoking, body mass index (BMI), total cholesterol, blood pressure, and fasting plasma glucose. Each metric is categorized into three levels—poor, intermediate, or ideal—based on clinical cut points, with an overall CVH status quantified by a score ranging from 0 to 14 (4, 6).

As research has progressed, the LS7 framework has been found to exhibit several limitations in the assessment of CVH, including insufficient sensitivity to individual heterogeneity, an incomplete representation of healthy dietary characteristics within the dietary metric, a lack of comprehensive consideration of health behaviors, and potential misclassification biases (7, 8). To address these shortcomings, the AHA has recently undertaken a comprehensive expansion of the CVH definition, introducing the “Life’s Essential 8” (LE8) framework. This updated model incorporates a new metric for sleep health and refines the quantification of the original indicators, adopting a continuous scoring system ranging from 0 to 100 to more accurately evaluate an individual’s cardiovascular well-being (7, 9). Subsequently, the work of López-Bueno et al. (10) has estimated the global prevalence of the eight key factors comprising the LE8, further corroborating the efficacy and utility of the LE8 framework in CVH assessment and laying the foundation for further exploration of the relationships between the LE8 components and CVH indicators.

The Atherogenic Index of Plasma (AIP) is a novel biomarker that quantifies an individual’s risk of atherosclerosis. It is calculated as the logarithm of the ratio of triglycerides (TG) to high-density lipoprotein cholesterol (HDL-C), with the aim of effectively predicting the occurrence and prognosis of CVD, as well as guiding clinical interventions (11–13). Previous research has demonstrated that the AIP is not only associated with the risk of CVD (14), but also correlates with various LE8 metrics, including BMI (15), Healthy Eating Index-2015 (HEI-2015) (16), physical activity (17), blood lipids (18), blood glucose (19), and blood pressure (20).

Despite the wealth of independent research on the LE8 and the AIP, the relationship between the two remains inadequately explored, particularly in terms of developing effective assessment models to elucidate the specific impacts of each LE8 component on the AIP. Therefore, this study conducted a comprehensive analysis utilizing data from the National Health and Nutrition Examination Survey (NHANES) in the United States, aiming to provide a more precise basis for CVD risk assessment, identify key lifestyle intervention targets, and ultimately enhance the decision-making capabilities of healthcare professionals in health management.

## 2 Methods

### 2.1 Data source and participants selection

The data were sourced from the NHANES database (https://wwwn.cdc.gov/nchs/nhanes/). NHANES, initiated by the Centers for Disease Control and Prevention, is a comprehensive survey utilizing questionnaires, laboratory tests, and physical examinations. The survey employs a multi-stage, stratified, and clustered probability sampling design to assess the health and nutritional status of a nationally representative sample of the U.S. civilian population, and to determine the prevalence and risk factors of major diseases. All study protocols have been approved by the National Center for Health Statistics Research Ethics Review Board, and written informed consent was obtained from all participants (21, 22).

This study initially included 59,842 participants from six NHANES cycles (2007–2018). After applying exclusion criteria, a total of 8,215 participants were included in the final analysis. Exclusion criteria comprised (1): insufficient data for calculating the AIP; (2) inadequate data for computing the LE8 score; (3) age below 20 years; (4) incomplete Patient Health Questionnaire-9 (PHQ-9) responses; (5) missing alcohol consumption data; (6) absent Poverty Income Ratio information; (7) lacking education level data; and (8) unknown marital status. (See **Supplementary Figure S1**).

### 2.2 Assessment of CVH by LE8 score

This study utilized the LE8 framework proposed by the AHA to assess CVH. The LE8 system integrated four health behavior metrics—diet, physical activity, nicotine exposure, and sleep—and four health factor metrics—BMI, blood lipids, blood glucose, and blood pressure. Each metric was evaluated on a 0-100 point scale. The overall LE8 score was calculated as the unweighted average of these eight metrics and was categorized into high CVH (80-100), moderate CVH (50-79), and low CVH (0-49) in accordance with AHA guidelines (7). Details of LE8 components are provided in **Supplementary Table S1**.

Diet quality was evaluated using the HEI-2015, which was calculated based on two 24-hour dietary recall surveys from participants and the USDA’s food pattern equivalents database. The HEI-2015 was a comprehensive tool comprising 13 components to assess dietary quality, with a maximum score of 100, where higher scores indicated healthier diets. Nine of these components focused on dietary adequacy (total fruits, whole fruits, total vegetables and legumes, whole grains, dairy, total protein foods, seafood and plant proteins, and fatty acids), while the remaining four assessed dietary moderation (intake of refined grains, sodium, added sugars, and saturated fats). Furthermore, to ensure the reliability and representativeness of blood pressure data, this study employed a standardized method of averaging three measurements. Detailed scoring criteria could be found in **Supplementary Table S2**.

### 2.3 Atherogenic index of plasma

The AIP was mathematically derived from the logarithm of the ratio of triglyceride to high-density lipoprotein cholesterol, both expressed in mmol/L, as follows: AIP=log_10_[TG (mmol/L)/HDL-C (mmol/L)] (11). Based on AIP values, participants were stratified into four risk categories: Extremely Low Risk Level (AIP < -0.3), Low Risk Level (-0.3 ≤ AIP < 0.1), Medium Risk Level (0.1 ≤ AIP < 0.24), and High Risk Level (AIP ≥ 0.24) (23).

### 2.4 Covariates

Covariates included age, gender (male and female), race (Mexican American, non-Hispanic White, non-Hispanic Black, and others), poverty-to-income ratio [categorized as low income (<1.3), medium income (1.3-3.5), and high income (≥3.5)], marital status (divorced/separated/widowed, married/living with partner, and never married), educational level (high school or less, some college, and college graduate or above), depression, and average daily alcohol consumption in the past 12 months.

### 2.5 Statistical analysis

NHANES employed design weights to ensure data representativeness. Since this study utilized data from two 24-hour dietary recalls, the specific weight “Dietary Two-Day Sample Weight (WTDR2D)” was applied according to NHANES guidelines. The WTDR2D weight was constructed based on the two-year cycle sample weight from the MEC and further adjusted for two key factors: (a) additional non-response and (b) distributional differences in dietary intake data collection by day of the week. Continuous variables were represented as means ± standard deviation, while categorical variables were presented as counts and percentages. Group differences were evaluated using a weighted linear regression model and a weighted chi-square test.

After adjusting for potential confounding factors, including age, gender, race, education level, marital status, poverty-to-income ratio, 12-month average daily alcohol consumption, and depression, a weighted ordinal logistic regression was employed to examine the association between the CVH level and the AIP risk level. The results were reported as adjusted odds ratios (ORs) with 95% confidence intervals (CIs). To test the proportional odds assumption—where a variable’s coefficients in the model’s different threshold equations are approximately the same or very similar, indicating that it satisfies the proportional odds assumption—a parallelism test was conducted. However, due to the large sample size, the parallelism test was overly sensitive, and even minor differences in coefficients could be statistically identified as violating the assumption. Therefore, this study employed the Wald test of the parallel lines assumption, and sequentially calculating the coefficients of each factor in the model, finding that the three models could be generally considered parallel.

All statistical analyses were performed using R software (version 4.3.2, The R Foundation; https://www.R-project.org) and STATA software (version 18, StataCorp; https://www.stata.com). A significance level of *P*<0.05 was considered statistically significant.

## 3 Result

### 3.1 General characteristics of the study population

**Table 1** presented the general characteristics of the study population based on different AIP risk levels derived from the NHANES 2007-2018 data. Highly significant differences (*P*<0.0001) were observed among participants across the different AIP risk levels in terms of age, gender, race, education level, marital status, poverty-to-income ratio, 12-month average daily alcohol consumption, depression, LE8 score, CVH level, health behaviors score, and health factors score. Compared to the lower AIP risk level groups, the high AIP risk level group was characterized by older age, higher male proportion, greater non-Hispanic White representation, predominantly married/living with a partner, lower educational level and reduced 12-month average daily alcohol consumption. Moreover, a notable gradient effect was observed between the LE8 score and the AIP risk level: the LE8 score decreased consistently as AIP risk increased. This trend was most pronounced in the Health Factors dimension, with scores declining from 82.52 in the extremely low AIP risk level group to 56.25 in the high AIP risk level group. However, certain indicators (physical activity, sleep health, and BMI) showed slight improvements in the high AIP risk level group, differing from the overall gradient trend.

**Table 1:** Weighted baseline characteristics of participants.

Characteristic	AIP Risk Level				P value
	Extremely Low Risk (AIP<-0.3)	Low Risk (-0.3 ≤ AIP < 0.1)	Medium Risk (0.1 ≤ AIP < 0.24)	High Risk (AIP ≥ 0.24)	
Age, years, Mean ± SE	45.85 ± 17.24	47.85 ± 17.40	49.27 ± 16.67	49.13 ± 14.79	<0.0001
Gender, n (%)					<0.0001
Male	721 (36.04)	479 (46.42)	1850 (50.15)	969 (64.82)	
Female	1279 (63.96)	552 (53.58)	1839 (49.85)	526 (35.18)	
Race, n (%)					<0.0001
Mexican American	114 (5.68)	82 (7.94)	429 (11.64)	155 (10.37)	
Non-Hispanic White	1276 (63.78)	724 (70.25)	2512 (68.09)	1085 (72.58)	
Non-Hispanic Black	323 (16.14)	102 (9.94)	192 (5.20)	66 (4.43)	
Others	288 (14.4)	122 (11.86)	556 (15.07)	189 (12.62)	
Education level, n (%)					<0.0001
High school or less	595 (29.76)	395 (38.29)	1609 (43.62)	645 (43.12)	
Some college	598 (29.90)	334 (32.35)	1200 (32.53)	457 (30.60)	
College graduate or above	807 (40.35)	303 (29.36)	880 (23.85)	393 (26.28)	
Marital status, n (%)					<0.0001
Divorced/Separated/Widowed	355 (17.73)	195 (18.93)	746 (20.23)	293 (19.63)	
Married/Living with a partner	1228 (61.40)	642 (62.30)	2406 (65.21)	998 (66.73)	
Never married	417 (20.86)	194 (18.77)	537 (14.56)	204 (13.64)	
Poverty-to-income ratio, n (%)					<0.0001
< 1.3	441 (22.06)	245 (23.74)	1083 (29.37)	409 (27.35)	
1.3–3.5	773 (38.66)	432 (41.89)	1452 (39.35)	582 (38.90)	
> 3.5	786 (39.28)	354 (34.38)	1154 (31.28)	505 (33.75)	
Depression, n (%)					<0.0001
No	1877 (93.84)	953 (92.45)	3242 (87.88)	1352 (90.46)	
Yes	123 (6.16)	78 (7.55)	447 (12.12)	143 (9.54)	
12-month average daily alcohol consumption, Mean ± SE	0.31 ± 0.63	0.24 ± 0.73	0.21 ± 0.75	0.18 ± 0.39	<0.0001
LE8 score, Mean ± SE	77.29 ± 13.19	68.39 ± 13.33	61.46 ± 13.64	59.59 ± 13.50	<0.0001
CVH level n (%)					<0.0001
Low (0–49)	54 (2.72)	93 (9.02)	755 (20.46)	359 (24.02)	
Moderate (50–79)	997 (49.83)	723 (70.13)	2646 (71.73)	1031 (68.99)	
High (80–100)	949 (47.45)	215 (20.85)	288 (7.81)	105 (6.99)	
Health behaviors score, Mean ± SE	72.06 ± 18.65	66.05 ± 19.42	62.10 ± 20.21	62.92 ± 19.54	<0.0001
HEI-2015 diet score, Mean ± SE	53.70 ± 12.30	50.41 ± 11.97	49.20 ± 11.66	49.23 ± 11.28	<0.0001
Diet score, Mean ± SE	48.12 ± 32.47	39.33 ± 31.69	36.40 ± 31.49	36.35 ± 30.76	<0.0001
Physical activity score, Mean ± SE	76.64 ± 39.19	69.17 ± 42.99	63.34 ± 45.24	66.73 ± 44.42	<0.0001
Nicotine exposure score, Mean ± SE	78.20 ± 35.13	72.66 ± 38.33	66.85 ± 39.92	65.32 ± 40.56	<0.0001
Sleep health score, Mean ± SE	85.27 ± 22.66	83.06 ± 24.94	81.83 ± 25.24	83.29 ± 23.82	0.0008
Health factors score, Mean ± SE	82.52 ± 15.86	70.72 ± 17.71	60.83 ± 17.89	56.25 ± 17.76	<0.0001
Body mass index score, Mean ± SE	76.99 ± 30.25	58.77 ± 33.54	46.19 ± 33.24	47.21 ± 30.69	<0.0001
Blood lipids score, Mean ± SE	84.46 ± 22.12	68.76 ± 28.16	54.94 ± 29.09	41.45 ± 28.87	<0.0001
Blood glucose score, Mean ± SE	90.64 ± 20.24	84.48 ± 24.07	77.16 ± 28.66	75.14 ± 30.49	<0.0001
Blood pressure score, Mean ± SE	77.98 ± 29.77	70.88 ± 32.54	65.01 ± 32.70	61.21 ± 32.98	<0.0001

AIP, Atherogenic index of Plasma; LE8, Life’s Essential 8; HEI, Healthy Eating Index; CVH, Cardiovascular Health.

### 3.2 Distribution relationship between the CVH level and the AIP risk level

**Figure 1:**
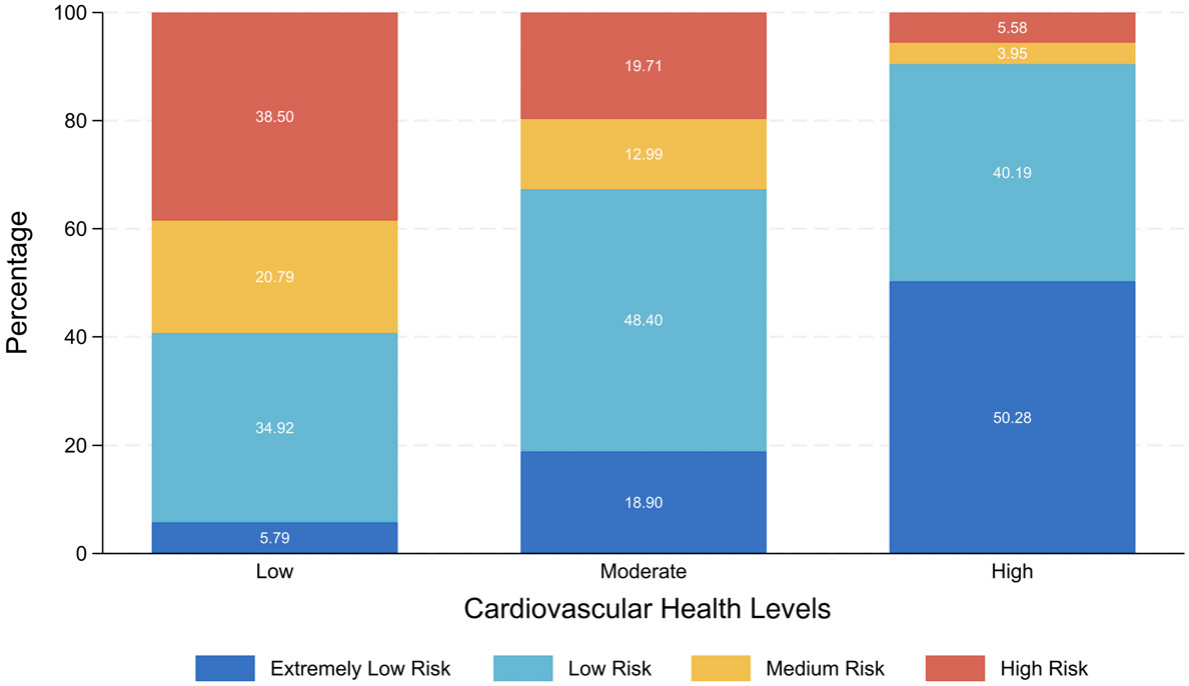
Stacked bar chart of the CVH level and the AIP risk level.

**Figure 1** presented as a stacked bar chart, illustrates the percentage distribution of AIP risk levels among individuals with different CVH levels. The result showed within the low CVH group, individuals at high AIP risk level comprised the largest proportion. As the LE8 score increased, the proportions of individuals at high and medium AIP risk levels steadily decreased, while those at extremely low and low AIP risk levels significantly rose. Most individuals with moderate CVH were classified within the low AIP risk level, whereas over 90% of individuals with high CVH fell into the extremely low or low AIP risk levels.

### 3.3 Association between the LE8 score and AIP risk levels

The weighted ordered logistic regression analysis results summarized in **Table 2** indicated that the LE8 score is significantly negatively associated with the AIP risk level. In the univariable analysis, every 10-point increase in the LE8 score was associated with an approximately 44% decrease in the probability of an individual moving up one AIP risk level (*OR*=0.56, *95%CI*: 0.53-0.58, *P*<0.001). Compared to the low CVH group, the probabilities of advancing one AIP risk level for the moderate and high CVH groups decreased by approximately 65% (*OR*=0.35, *95%CI*: 0.29-0.41, *P*<0.001) and 92% (*OR*=0.08, *95%CI*: 0.06-0.10, *P*<0.001), respectively. This finding was further confirmed in the multivariable analysis, which showed that even after adjusting for age, gender, race, poverty-to-income ratio, education level, marital status, depression, and average daily alcohol consumption, the LE8 score remained an independent predictor of the AIP risk level. In the multivariable analysis, for every 10-point increase in the LE8 score, the probability of an individual moving up one AIP risk level decreased by approximately 49% (*OR*=0.51, *95%CI*: 0.49-0.54, *P*<0.001). Compared to the low CVH group, the probabilities of advancing one AIP risk level decreased by approximately 69% for the moderate CVH group (*OR*=0.31, *95%CI*: 0.26-0.37, *P*<0.001) and by 93% for the high CVH group (*OR*=0.07, *95%CI*: 0.05-0.09, *P*<0.001). Additionally, both the health behavior score and the health factors score exhibited a significant negative correlation with the AIP risk level. In the univariable ordinal logistic regression analysis, each 10-point increase in the health behaviors score was associated with a 15% decrease in the probability of an individual advancing one AIP risk level (*OR*=0.85, *95%CI*: 0.83-0.88, *P*<0.001). For health factors scores, this probability decreased by approximately 39% (*OR*=0.61, *95%CI*: 0.59-0.63, *P*<0.001). In the multivariable ordinal logistic regression analysis, each 10-point increase in the health behaviors score corresponded to a 14% decrease in the probability of advancing one AIP risk level (*OR*=0.86, *95%CI*: 0.83-0.89, *P*<0.001). For health factors scores, the probability decreased by approximately 45% (*OR*=0.55, *95%CI*: 0.53-0.57, *P*<0.001).

**Table 2:** Association between LE8 and AIP.

Variables	Univariable model		Multivariable model	
	OR (95% CIs)	P value	OR (95% CIs)	P value
LE8 score				
Per 10-point increase	0.56 (0.53, 0.58)	< 0.001	0.51 (0.49, 0.54)	< 0.001
Low CVH (0–49)	Reference		Reference	
Moderate CVH (50–79)	0.35 (0.29, 0.41)	< 0.001	0.31 (0.26, 0.37)	< 0.001
High CVH (80–100)	0.08 (0.06, 0.10)	< 0.001	0.07 (0.05, 0.09)	< 0.001
Health behaviors score				
Per 10-point increase	0.85 (0.83,0.88)	< 0.001	0.86 (0.83, 0.89)	< 0.001
Low (0–49)	Reference		Reference	
Moderate (50–79)	0.73 (0.63, 0.84)	< 0.001	0.75 (0.64, 0.89)	< 0.001
High (80–100)	0.46 (0.38, 0.55)	< 0.001	0.50 (0.41, 0.60)	< 0.001
Health factors score				
Per 10-point increase	0.61 (0.59, 0.63)	< 0.001	0.55 (0.53, 0.57)	< 0.001
Low (0–49)	Reference		Reference	
Moderate (50–79)	0.36 (0.31, 0.43)	< 0.001	0.32 (0.27, 0.38)	< 0.001
High (80–100)	0.08 (0.07, 0.10)	< 0.001	0.06 (0.05, 0.08)	< 0.001

OR, Odds Ratio; CIs, Confidence Intervals; CVH, Cardiovascular Health; LE8,Life’s Essential 8.

Univariable model: unadjusted model.

Multivariable model: adjusted for age, gender, race, poverty-to-income ratio, education levels, marital status, depression, and 12-month average daily alcohol consumption.

**Figure 2:**
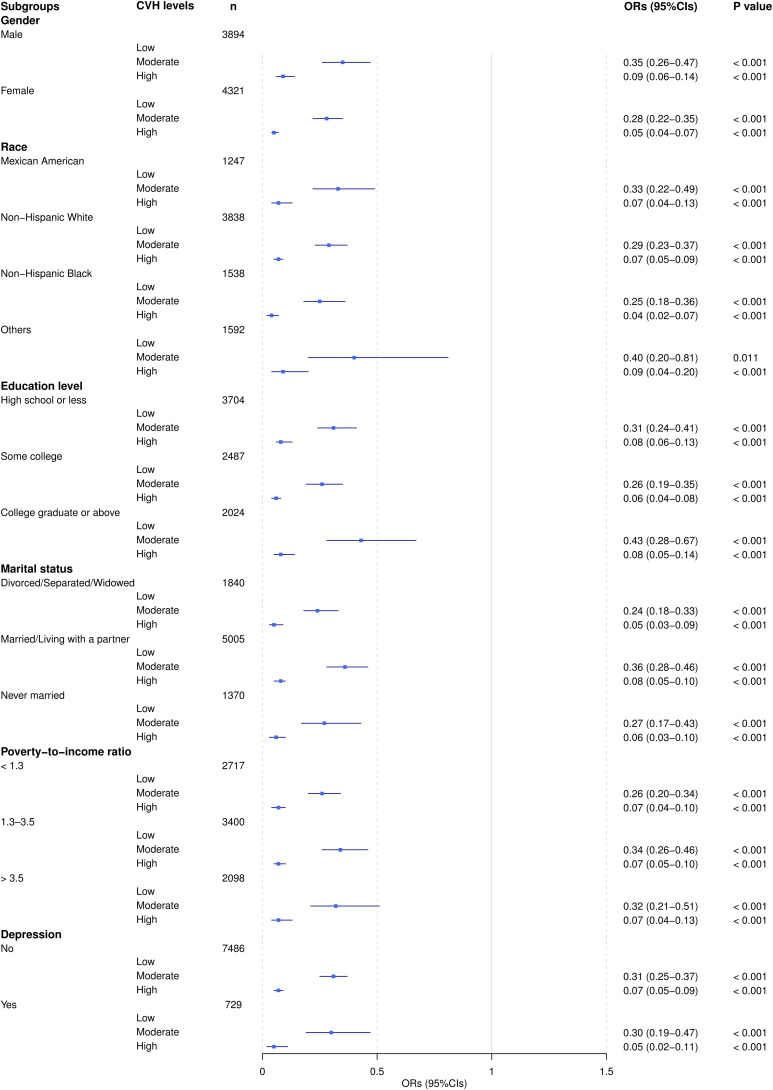
Adjusted ORs (95% CI) of AIP by CVH level subgroups. ORs were adjusted for all listed covariates except the stratification variable in each subgroup analysis.

**Figure 2** illustrated the adjusted ORs and 95% CIs for the association between the CVH level and the AIP risk level across different subgroups. The result indicated that, in all subgroups, an increase in the CVH level was significantly associated with a reduction in the AIP risk level. This trend was validated across various subgroups defined by gender, race, education level, marital status, poverty-to-income ratio, and depression symptoms. Notably, the reduction in AIP risk level associated with a higher CVH was particularly pronounced among females, non-Hispanic Black individuals, and individuals with depression.

### 3.4 Association between individual LE8 components and AIP

**Figure 3:**
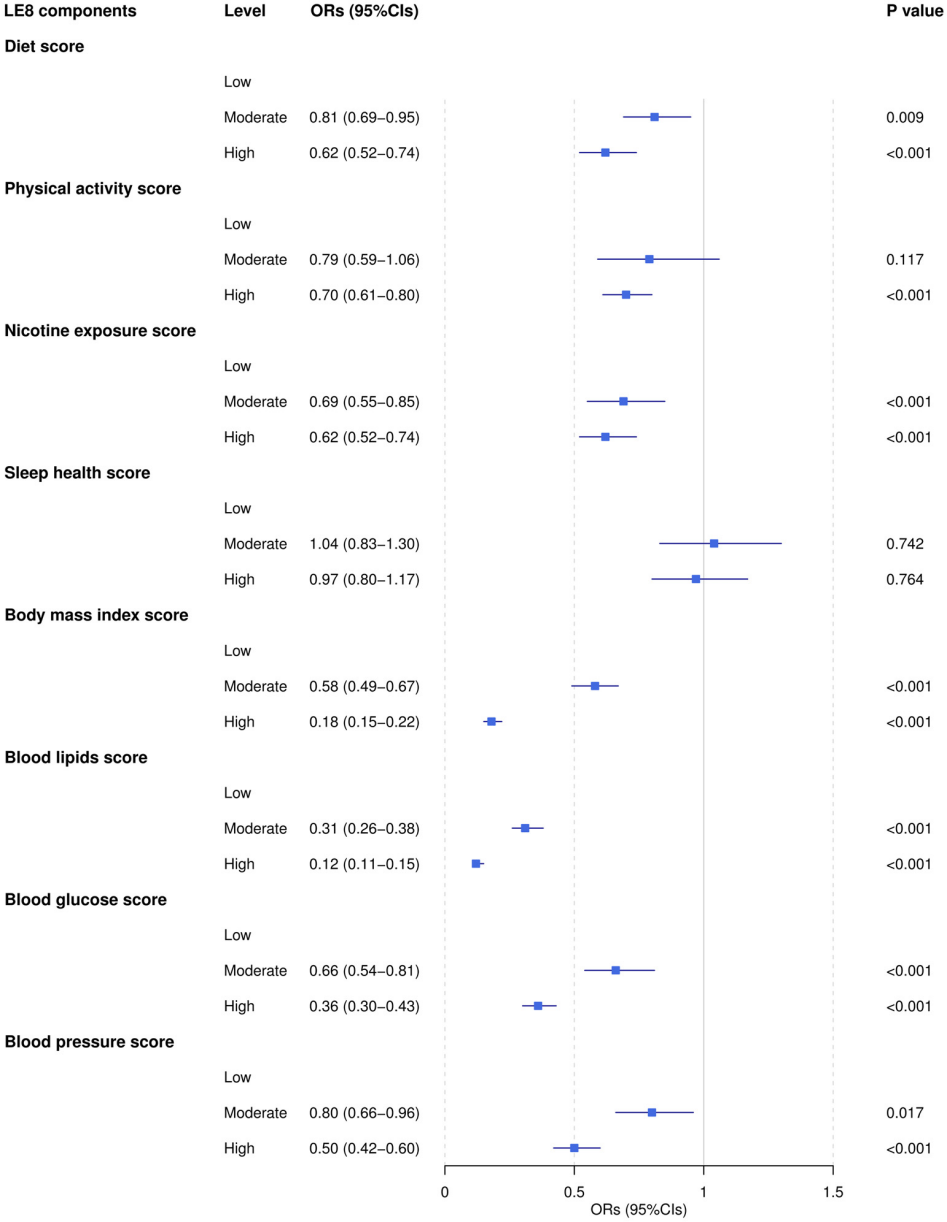
The association between LE8 components and AIP. ORs were adjusted for age, gender, race, poverty-to-income ratio, education levels, marital status, depression, and 12-month average daily alcohol consumption.

The result presented in **Figure 3** illustrates the adjusted ORs and 95% CIs for the association between different levels (low, moderate, high) of LE8 components and the AIP risk level. The finding indicated that, with the exception of sleep health, improvements in all other LE8 components were significantly associated with a reduction in the AIP risk level, with the most notable effects observed for improvements in BMI and blood lipids scores. Notably, the high physical activity score was significantly associated with lower AIP risk levels (*OR*=0.70, *95%CI*: 0.61-0.80, *P*<0.001). In contrast, the result for moderate physical activity scores did not achieve statistical significance (*OR*=0.79, *95%CI*: 0.59-1.06, *P*=0.117).

## 4 Discussion

To our knowledge, this study was the first to comprehensively explore the relationship between CVH and AIP within the framework of LE8 using data from the NHANES database. The findings indicated a significant negative correlation between the LE8 score and the AIP risk level: higher LE8 scores were associated with lower AIP risk levels. Individuals with high CVH predominantly fell within the extremely low or low AIP risk levels, whereas those with low CVH were more frequently situated in the high AIP risk level. Furthermore, improvements in specific LE8 components were correlated with reductions in AIP risk level, particularly notable were the effects of enhancements in BMI and blood lipid levels.

This study identified a negative correlation between the LE8 score and the AIP risk level, consistent with the findings of Cunha et al. (24), which reinforces the idea that maintaining a healthy lifestyle is crucial for reducing CVD risk. A meta-analysis conducted by Sebastian et al. (25) revealed that individuals with high LE8 scores experienced lower all-cause mortality rate (*HR*=0.54, *95%CI*: 0.43-0.69, *P*<0.001) and disease-specific mortality rate (*HR*=0.37, *95%CI*: 0.26-0.52, *P*<0.001). It can be attributed to the LE8 score’s comprehensive assessment of multiple health lifestyle factors, which enhances the development of personalized prevention and treatment strategies, making it a valuable protective factor against CVD risk (7, 9, 10). Conversely, AIP is regarded as a sensitive predictor of CVD risk and has demonstrated excellent performance in identifying CVD risk factors: the area under the receiver operating characteristic curve was 0.909 (*P*<0.001), with an optimal cut-off value of 0.468, and high sensitivity (84.80%) and specificity (78.60%) (26). When the LE8 score, serving as a protective factor against CVD risk, demonstrates a negative correlation with the AIP, a sensitive predictor of CVD risk, this relationship potentially implies that individuals can actively modulate their metabolic characteristics through lifestyle optimization, thereby effectively mitigating CVD risk. Furthermore, despite the differences between the LE8 and LS7 frameworks, the CVH level remained significantly negatively correlated with AIP within the LS7 framework (27, 28). This suggests that the negative correlation between CVH and AIP exhibits strong stability and universality, thereby enhancing the reliability of CVH as indicators for the prevention and management of CVD risk.

This study revealed that, with the exception of sleep health indicators, the remaining seven LE8 metrics—namely diet, physical activity, nicotine exposure, BMI, blood lipids, blood glucose, and blood pressure—exhibited significant correlations with the AIP risk level. Notably, the improvements in BMI and blood lipid levels were particularly pronounced. Research conducted by Chang et al. (28) similarly confirmed the associations of these seven metrics with AIP and indicated that BMI exerted the most substantial influence on AIP (*PR*=3.76; *95%CI*: 3.27-4.32; *P*<0.001). Analogously, the logistic regression analysis conducted by Shen et al. (29) revealed that smoking status, BMI, physical activity, sodium intake, total cholesterol, blood pressure, and fasting blood glucose all exhibited significant impacts on AIP (*P*<0.05), with BMI demonstrating the strongest correlation with total cholesterol and AIP. This may be attributed to the fact that elevated BMI is typically associated with abnormal or excessive fat accumulation, particularly in the visceral adipose tissue. Visceral fat is closely linked to metabolic syndrome (as increased visceral fat releases free fatty acids into the portal vein, becoming the primary source of hepatic TG synthesis, leading to elevated TG levels), insulin resistance (the rise in free fatty acids can suppress glucose transport or phosphorylation in muscle, thereby inducing insulin resistance), and inflammatory responses (the increased secretion of factors such as tumor necrosis factor-α and interleukin-6 leads to dysregulation of adipokine secretion), all of which contribute to elevated TG and reduced HDL-C, directly resulting in an increased AIP (30–33). Furthermore, previous research has revealed that obese adolescents exhibit significantly higher AIP risk levels compared to healthy controls, and a positive correlation between AIP and BMI has also been observed in adolescents with non-alcoholic fatty liver disease (34). However, our study only included participants aged 20 years and above; future investigations would benefit from expanding the age range or focusing solely on the younger population to further elucidate the dynamic changes in the relationship between BMI and AIP across different age groups.

Within the LE8 framework, sleep health is a multidimensional construct encompassing duration, timing, regularity, efficiency, satisfaction, and impact on daytime alertness. This metric is closely associated with CVD, mental health, and social determinants, and is of paramount importance for overall health management (7). A meta-analysis conducted by Behnoush et al. (35) on three composite lipid indices—AIP, lipid accumulation product, and visceral adiposity index—in patients with obstructive sleep apnea (OSA) revealed that AIP was significantly elevated in OSA patients, with the highest AIP values observed in those with severe OSA. This may be attributed to the fact that OSA patients, due to respiratory pauses and hypoxic events, are unable to obtain sufficient deep sleep, leading to inadequate sleep duration, which in turn triggers insulin resistance, elevated TG levels, and reduced HDL-C, ultimately resulting in an increase in AIP. However, our study found no statistically significant relationship between sleep health and AIP risk level. This discrepancy may be attributed to the fact that this study primarily relied on participants’ self-reported average nightly sleep duration to calculate the sleep health score, which may have failed to comprehensively capture the multidimensional nature of sleep health. Future research should employ more comprehensive sleep health assessment metrics and incorporate objective monitoring devices, such as polysomnography or wearable technology, to record multiple dimensions of sleep duration, quality, and efficiency. This approach would further validate the relationship between sleep health and AIP risk level, providing robust intervention strategies for CVH management.

Interestingly, the findings of this study revealed that the moderate-level physical activity score, based on self-reported minutes of moderate or vigorous physical activity (MVPA) per week, did not show statistical significance when compared to the low-level physical activity score (*P*=0.117). In contrast, the high-level physical activity score demonstrated statistical significance compared to the low-level score (*P*<0.001). However, previous research has shown a significant inverse dose-response relationship between physical activity and AIP: when MVPA was in the Q1 range of 0 MET·minutes/month, AIP exhibited a slight increase; when MVPA was in the Q2 range of 444-491 MET·minutes/month, AIP remained relatively unchanged; when MVPA was in the Q3 range of 2,457-2,596 MET·minutes/month, AIP decreased significantly; and when MVPA was in the Q4 range of 12,711-14,835 MET·minutes/month, AIP exhibited the most substantial reduction (17). Additionally, Edwards et al. (36) conducted further research, revealing that the negative correlation between physical activity and AIP was entirely mediated by central obesity: without considering central obesity, MVPA was significantly associated with reduced odds of high AIP (*OR*=0.58, *95%CI*: 0.41-0.82, *P*=0.004); Yet, when central obesity was included as a covariate in the model, the association between MVPA and AIP was no longer significant (*OR*=0.80, *95%CI*: 0.55-1.18, *P*=0.260). In summary, the result of our study suggested that moderate-level physical activity may not have reached the critical threshold necessary to improve the dose-response relationship between central obesity and AIP, which may explain the lack of significant difference compared to low-level physical activity. Conversely, high-level physical activity appear to effectively improve central obesity, thereby significantly reducing AIP risk levels. Future research should focus on identifying the optimal types and durations of physical activity that can enhance central obesity and AIP, providing a solid foundation for the development of more targeted and effective health management strategies.

This study had several limitations. First, the cross-sectional design of the study precluded the determination of causal relationships, and therefore, it could not be concluded that the improvement in the LE8 score directly led to the reduction in AIP risk level. Second, while the study adjusted for multiple confounding factors, there may have been potential unmeasured confounding variables that could have influenced the interpretation of the results. Third, some of the data — such as smoking, alcohol consumption, and dietary intake — relied on self-reports by the participants, which could have introduced information bias and affected the accuracy of the data. Fourth, the conversion of continuous variables into ordinal variables in this study may have resulted in the loss of information and reduction of details. Additionally, the determination of classification criteria involved a certain degree of subjectivity, which could have led to the incorrect classification of some individuals near the classification boundaries. Finally, this study primarily focused on AIP as an indicator of CVD risk, and it may have overlooked the comprehensive application of other relevant indicators and assessment methods.

## 5 Conclusion

Our study demonstrated that the LE8 exhibits a significant negative correlation with the AIP, a key predictive marker for CVD risk. This correlation underscores the critical role of CVH level in mitigating AIP risk level and highlights the essential importance of lifestyle interventions in CVD prevention. With the exception of sleep health, improvements in the components of LE8 — particularly BMI and blood lipid levels — were strongly associated with reductions in AIP risk levels. These findings not only validate the effectiveness of the LE8 as a protective factor against CVD risk but also emphasize its potential application in individual health management, thereby providing a robust theoretical foundation for precision medicine. Future research should further investigate the relationship between sleep health and the AIP to enhance comprehensive health management strategies.

## Data Availability

The original contributions presented in the study are included in the article/**Supplementary Material**. Further inquiries can be directed to the corresponding author.
