# Supplementary material for: Association between Life’s Essential 8 and Atherogenic Index of Plasma in adults: insights from NHANES 2007–2018
Source: Front Endocrinol (Lausanne). 2025 Feb 18;16:1506884. doi: 10.3389/fendo.2025.1506884 (PMC11876005; doi:10.3389/fendo.2025.1506884)
Supplement: Supplementary file 1 [file DataSheet1.docx]

Supplementary Material


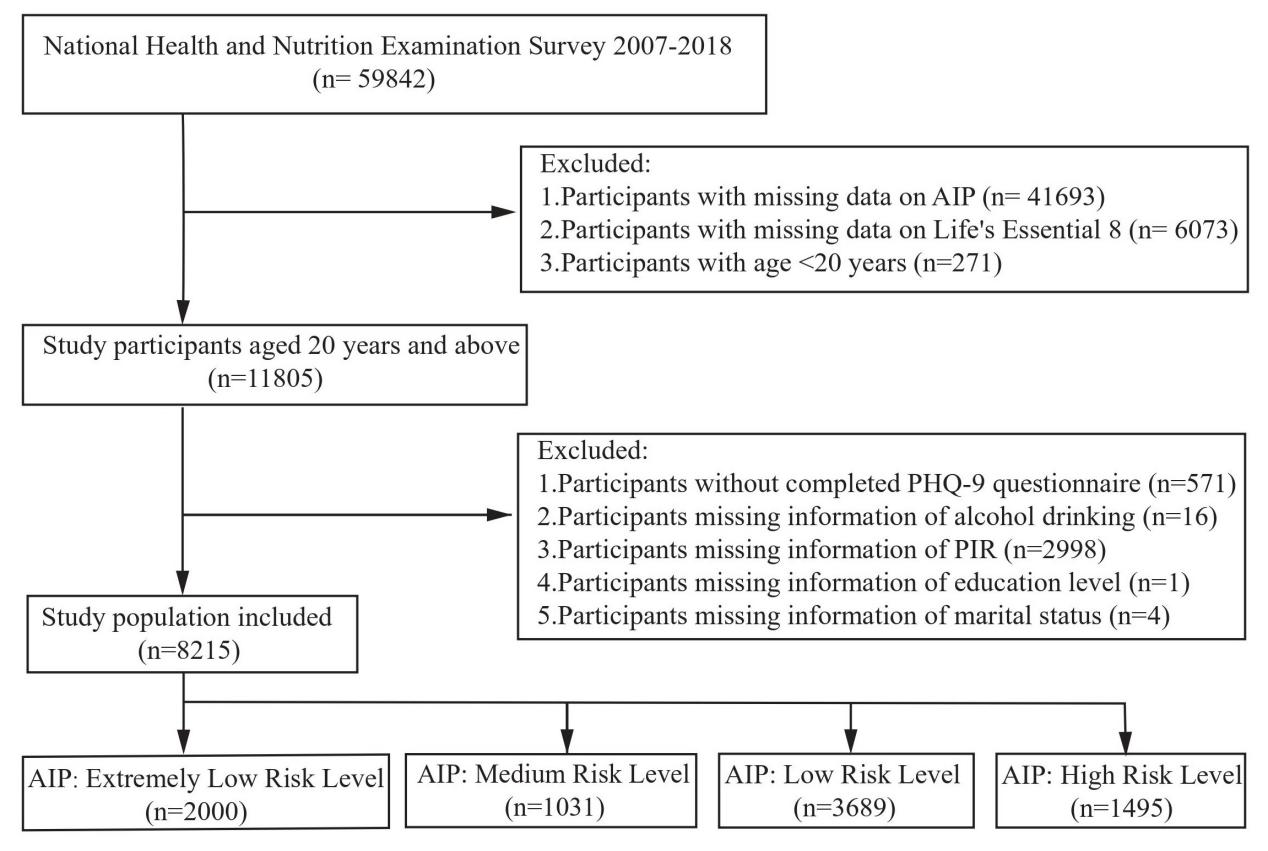


**Supplementary Figure 1.** Flowchart of the sample selection from NHANES 2007-2018.

**Supplementary Table 1.** Definition and scoring approach for the American Heart Association’s Life’s Essential 8 score.

| Domain | CVH Metric | Measurement | Quantification of CVH Metric | |
| --- | --- | --- | --- | --- |
|  |  |  | Scoring | |
|  |  |  | Points | Quantile/Minutes/Status/Level |
| Health behaviors | Diet | Healthy Eating Index-2015 diet score percentile | 100 | ≥95th percentile (top/ideal diet) |
|  |  |  | 80 | 75th-94th percentile |
|  |  |  | 50 | 50th-74th percentile |
|  |  |  | 25 | 25th-49th percentile |
|  |  |  | 0 | 1st-24th percentile  (bottom/least ideal quartile) |
|  | Physical activity | Self-reported minutes of moderate or vigorous physical activity per week | 100 | ≥150 |
|  |  |  | 90 | 120–149 |
|  |  |  | 80 | 90–119 |
|  |  |  | 60 | 60–89 |
|  |  |  | 40 | 30–59 |
|  |  |  | 20 | 1–29 |
|  |  |  | 0 | 0 |
|  | Nicotine exposure | Self-reported use of cigarettes or inhaled nicotine-delivery system | 100 | Never smoker |
|  |  |  | 75 | Former smoker, quit ≥5 y |
|  |  |  | 50 | Former smoker, quit 1–<5 y |
|  |  |  | 25 | Former smoker, quit <1 y, or currently using inhaled NDS |
|  |  |  | 0 | Current smoker |
|  |  |  | Subtract 20 points (unless score is 0) for living with active indoor smoker in home | |
|  | Sleep health | Self-reported average hours of sleep per night Example tools for measurement: “On average, how many hours of sleep do you get per night?” | 100 | 7–<9 |
|  |  |  | 90 | 9–<10 |
|  |  |  | 70 | 6–<7 |
|  |  |  | 40 | 5–<6 or ≥10 |
|  |  |  | 20 | 4–<5 |
|  |  |  | 0 | <4 |
| Health factors | BMI | Body weight (kilograms) divided by height squared (meters squared) | 100 | <25 |
|  |  |  | 70 | 25.0–29.9 |
|  |  |  | 30 | 30.0–34.9 |
|  |  |  | 15 | 35.0–39.9 |
|  |  |  | 0 | ≥40.0 |
|  | Blood lipids | Plasma total and HDL cholesterol with calculation of non–HDL cholesterol | 100 | <130 |
|  |  |  | 60 | 130–159 |
|  |  |  | 40 | 160–189 |
|  |  |  | 20 | 190–219 |
|  |  |  | 0 | ≥220 |
|  |  |  | If drug-treated level, subtract 20 points | |
|  | Blood glucose | Fasting blood glucose (FBG) or casual HbA1c | 100 | No history of diabetes and FBG <100 (or HbA1c <5.7) |
|  |  |  | 60 | No diabetes and FBG 100–125 (or HbA1c 5.7–6.4) (prediabetes) |
|  |  |  | 40 | Diabetes with HbA1c <7.0 |
|  |  |  | 30 | Diabetes with HbA1c 7.0–7.9 |
|  |  |  | 20 | Diabetes with HbA1c 8.0–8.9 |
|  |  |  | 10 | Diabetes with Hb A1c 9.0–9.9 |
|  |  |  | 0 | Diabetes with HbA1c ≥10.0 |
|  | Blood pressure | Appropriately measured systolic and diastolic Blood pressure | 100 | <120/<80 (optimal) |
|  |  |  | 75 | 120–129/<80 (elevated) |
|  |  |  | 50 | 130–139 or 80–89 (stage 1 hypertension) |
|  |  |  | 25 | 140–159 or 90–99 |
|  |  |  | 0 | ≥160 or ≥100 |
|  |  |  | Subtract 20 points if treated level | |

**Supplementary Table 2.** Healthy Eating Index-2015 components & scoring standards.

| Component | Maximum points | Standard for maximum score | Standard for minimum score of zero |
| --- | --- | --- | --- |
| Adequacy |  |  |  |
| Total Fruits | 5 | ≥0.8 c equivalents/1,000 kcal | No fruit |
| Whole Fruits | 5 | ≥0.4 c equivalents/1,000 kcal | No whole fruit |
| Total Vegetables | 5 | ≥1.1 c equivalents/1,000 kcal | No vegetables |
| Greens and Beans | 5 | ≥0.2 c equivalents/1,000 kcal | No dark green vegetables or beans and peas |
| Whole Grains | 10 | ≥1.5 oz equivalents/1,000 kcal | No whole grains |
| Dairy | 10 | ≥1.3 c equivalents/1,000 kcal | No dairy |
| Total Protein Foods | 5 | ≥2.5 oz equivalents/1,000 kcal | No protein foods |
| Seafood and Plant Proteins | 5 | ≥0.8 c equivalents/1,000 kcal | No seafood or plant proteins |
| Fatty Acids | 10 | (PUFAs^a^+MUFAs^b^)/SFAs^c^ ≥2.5 | (PUFAs+MUFAs)/SFAs ≤1.2 |
| Moderation |  |  |  |
| Refined Grains | 10 | ≤1.8 oz equivalents/1,000 kcal | ≥4.3 oz equivalents/1,000 kcal |
| Sodium | 10 | ≤1.1 g/1,000 kcal | ≥2.0 g/1,000 kcal |
| Added Sugars | 10 | ≤6.5% of energy | ≥26% of energy |
| Saturated Fats | 10 | ≤8% of energy | ≥16% of energy |

^a^PUFAs=polyunsaturated fatty acids; ^b^MUFAs=monounsaturated fatty acids; ^c^SFAs=saturated fatty acids.
